# Is prehospital endobronchial intubation a risk factor for subsequent ventilator associated pneumonia? A retrospective analysis

**DOI:** 10.1371/journal.pone.0217466

**Published:** 2019-05-23

**Authors:** Ana Catalina Hernandez Padilla, Timothée Trampont, Thomas Lafon, Thomas Daix, Dominique Cailloce, Olivier Barraud, François Dalmay, Philippe Vignon, Bruno François

**Affiliations:** 1 INSERM CIC 1435, CHU Dupuytren, Limoges, France; 2 Service d’Accueil des Urgences, CHU Dupuytren, Limoges, France; 3 Réanimation polyvalente, CHU Dupuytren, Limoges, France; 4 INSERM UMR 1092, Université Limoges, Limoges, France; 5 SAMU/SMUR, CHU Dupuytren, Limoges, France; 6 Laboratoire de Bactériologie–Virologie–Hygiène, CHU Dupuytren, Limoges, France; 7 INSERM UMR 1094, Université Limoges, Limoges, France; San Gerardo Hospital, ITALY

## Abstract

More than half of patients under mechanical ventilation in the intensive care unit (ICU) are field-intubated, which is a known risk factor for ventilator associated pneumonia (VAP). We assessed whether field endobronchial intubation (EBI) is associated with the development of subsequent VAP during the ICU stay. This retrospective, nested case-control study was conducted in a cohort of field-intubated patients admitted to an ICU of a teaching hospital during a three-year period. Cases were defined as field-intubated patients with EBI and controls corresponded to field-intubated patients with proper position of the tracheal tube on admission chest X-ray. Primary endpoint was the development of early VAP. Secondary endpoints included the development of early ventilator associated tracheo-bronchitis, late VAP, duration of mechanical ventilation, length of stay and mortality in the ICU. A total of 145 patients were studied (mean age: 54 ± 19 years; men: 74%). Reasons for field intubation were predominantly multiple trauma (49%) and cardiorespiratory arrest (38%). EBI was identified in 33 patients (23%). Fifty-three patients (37%) developed early or late VAP. EBI after field intubation was associated with a nearly two-fold increase of early VAP, though not statistically significant (30% vs. 17%: p = 0.09). No statistically significant difference was found regarding secondary outcomes. The present study suggests that inadvertent prehospital EBI could be associated with a higher incidence of early-onset VAP. Larger studies are required to confirm this hypothesis. Whether strategies aimed at decreasing the incidence and duration of EBI could reduce the incidence of subsequent VAP remains to be determined.

## Introduction

Ventilator associated pneumonia (VAP) is the most frequent infectious complication in the intensive care unit (ICU) which is associated with increased mortality, ICU length of stay and hospital costs [1–4]. Field intubation has been reported as an independent risk factor for VAP [1,5–7]. Nevertheless, it is often necessary and accounts for 70 to 77% of tracheal intubation in ventilated trauma patients admitted to the ICU [5,8].

Endobronchial intubation (EBI) is a complication of field intubation in 11 to 21% of patients [9], and is attributed to limited equipment and space, poor lighting, and difficult environment to perform advanced procedures [10]. EBI can be unrecognized by the physicians despite careful clinical examination when relying only on lung auscultation [11], which can be misleading in two thirds of the cases and is highly operator dependent [11,12]. Duration of EBI and its correction following in-hospital assessment using either chest X-ray [13–15], ultrasound [16–18], or even a flexible fiberscope [19] could be time-consuming and requires 30 minutes to several hours [15]. EBI can lead to over-inflation of the intubated lung and partial-to-complete atelectasis of the contralateral lung [12,20], and may therefore be associated with an increased risk of subsequent VAP. Accordingly, we assessed whether initial EBI following a field intubation increases the risk of developing early VAP in these patients who are subsequently hospitalized in the ICU. We also explored the potential relationship between EBI and early ventilator associated tracheo-bronchitis (VAT) and late VAP. Esophageal intubation which is another complication of field intubation was out of scope of the present study.

## Methods

### Study design and data collection

This single-center, retrospective, nested case-control, observational study was conducted between January 2012 and December 2015 and was approved by the Ethical Committee of Limoges University Hospital that waived the need for informed consent due to its observational nature. The study included all consecutive patients ≥ 18 years-old who underwent emergency field intubation, defined as the need for immediate tracheal intubation during the initial prehospital management, in a context of cardiac arrest, acute respiratory failure or decreased level of consciousness related or not to severe head trauma. Patients were excluded if they had documented or suspected pneumonia, or overt aspiration at the time of admission as assessed during direct laryngoscopy by the pre-hospital resuscitation team, if they were extubated within 48 hours after hospital admission, if underlying diseases made the interpretation of chest X-ray difficult for VAP diagnosis or tube positioning (e.g., lung surgery, lung neoplasia and severe pulmonary edema), or if they died within the first 2 days of ICU stay. All ventilated patients were admitted to our medical-surgical ICU after hospital admission. A bundle of VAP prophylaxis was uniformly applied for each patient according to current international recommendations (elevation of patient’s head of bed, daily sedation interruption and daily assessment of readiness for extubation, peptic ulcer prevention and deep venous thrombosis prophylaxis) [21].

The primary endpoint was the development of early VAP (i.e., ≤ day 4) and secondary endpoints were the development of other respiratory tract infections (early VAT, late VAP, late VAT), duration of mechanical ventilation, length of stay and mortality in the ICU.

For each patient, the following variables were recorded: sex, age, comorbidities, simplified acute physiology score (SAPS) 2 on admission, reason for field intubation, duration of EBI estimated as the time lag between departure of the prehospital medical team from the scene (registered in the pre-hospital medical records) and the first chest X-ray performed systematically at the time of hospital admission, VAT or VAP occurrence and date of diagnosis, microbiological documentation, use of antibiotics within the last month and for reasons different from VAP in the first 7 days following admission, fluid balance and transfusions at day 4 and day of VAP diagnosis when applicable, ICU length of stay and mortality. Data were collected directly from chart review of electronic medical records (ICCA Philips, Suresnes, France).

The diagnosis of EBI relied on a concordant identification by two experienced intensivists [TD and BF] based on their independent interpretation of the first chest X-ray performed immediately after hospital admission. EBI was defined by the right-sided location of the tube into the main bronchus or less than 10 mm above the carina. Physicians assessing the EBI did not participate in the adjudication committee that evaluated the development of VAT or VAP during ICU stay.

### VAP and VAT definitions

VAP was diagnosed by an independent adjudication committee composed by two experienced intensivists [TD and TL]. Adjudicators had neither access to the first chest X-ray performed to ascertain the position of the tracheal tube nor to its interpretation. VAP was defined as a pneumonia event in a patient intubated with an endotracheal or nasotracheal tube and receiving positive pressure ventilation support. Patients were diagnosed with pneumonia when fitting all three radiologic, clinical and microbiological criteria as follows: *Radiologic criteria*: new or worsening infiltrate consistent with pneumonia on chest X-ray obtained within 24h of the onset of the symptoms; *Clinical criteria*: at least 1 sign of systemic inflammation and 2 minor criteria (systemic signs of infection, purulent endotracheal secretions, and/or auscultation findings of pneumonia/consolidation) or one or more major criteria (Acute changes in ventilator support system to enhance oxygenation, either a drop in the PaO2/FiO2 below 240mmHg or a sustained decrease of > 50 mmHg for at least 4 hours); *Microbiological criteria*: at least 1 positive culture obtained within 24 hours of the onset of the pneumonia event, using an endotracheal aspiration, bronchoalveolar lavage, protected distal sampling, pleural fluid aspirate, lung tissue culture, or positive blood culture without extra-pulmonary source of infection [22]. Onset of VAT was noted and was defined using all of the following criteria: fever (>38°C) with no other recognizable cause, purulent sputum production, positive endotracheal aspirate culture yielding a new bacteria, and no radiographic signs of new pneumonia [4,23,24].

The cutoff defining occurrence of early versus late VAP was set on day 4 [4].

### Statistical analysis

All analyses were performed using the SAS 9.1.3 software (SAS Institute, Cary, USA). Data are displayed using descriptive statistics: total numbers and percentages were used for categorical variables and median and inter-quartile range (IQR) for continuous variables considering their high dispersion. Categorical variables were compared between patients with EBI and those with adequate tracheal tube position using the Chi^2^ or Fisher exact test as appropriate, while continuous variables were compared using the Student’s t-test. Pearson correlation test was used to assess the potential relation between EBI duration and the development of VAP. Statistical significance was defined as a *p* value <0.05. A stepwise multivariate logistic regression analysis was performed to identify independent variables associated with the development of early VAP. All variables with a *p* value < 0.20 in the univariate analysis were incorporated into the model (S1 Table).

## Results

**Fig 1:**
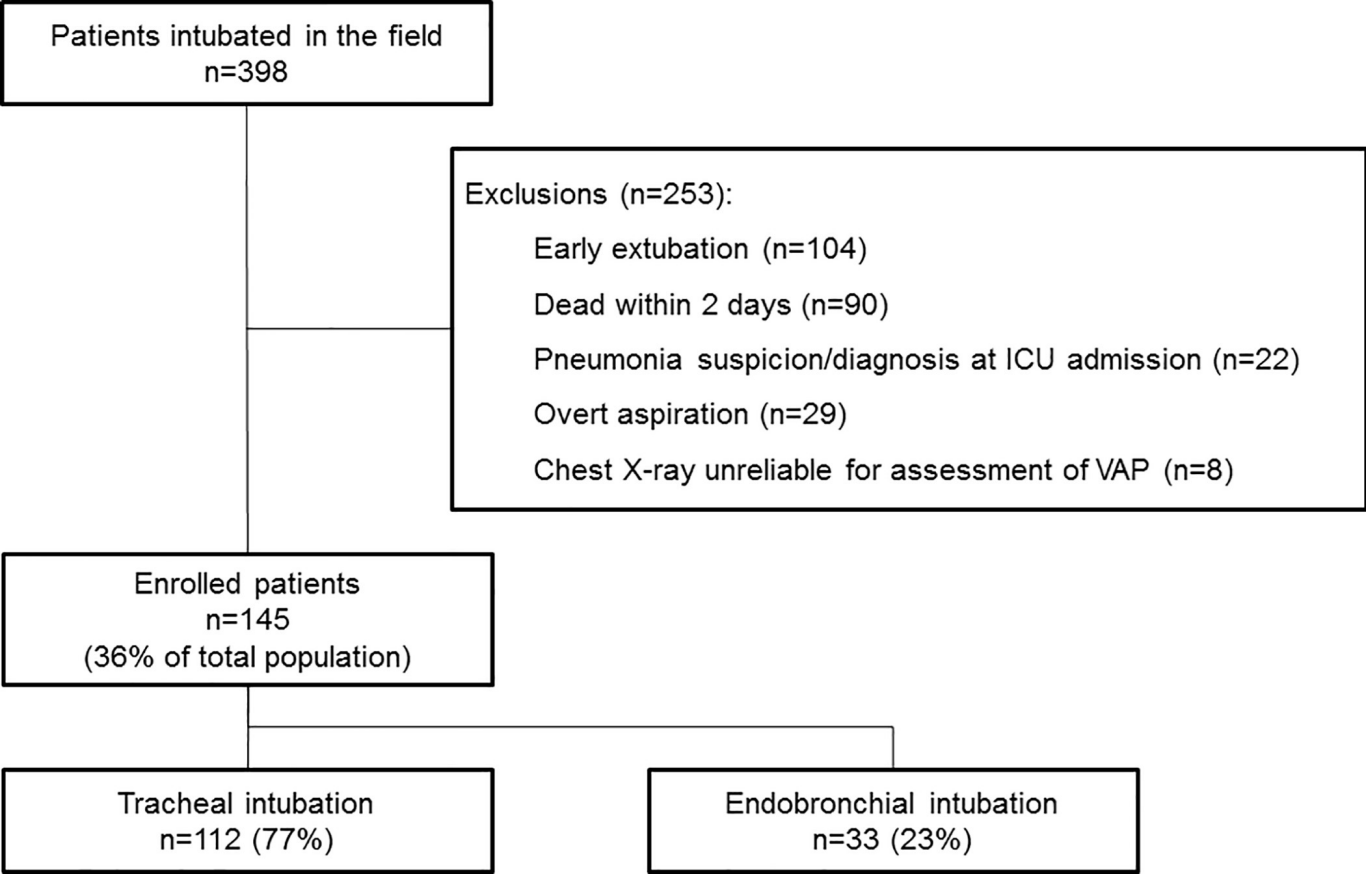
Flow chart of the study.

**Fig 2:**
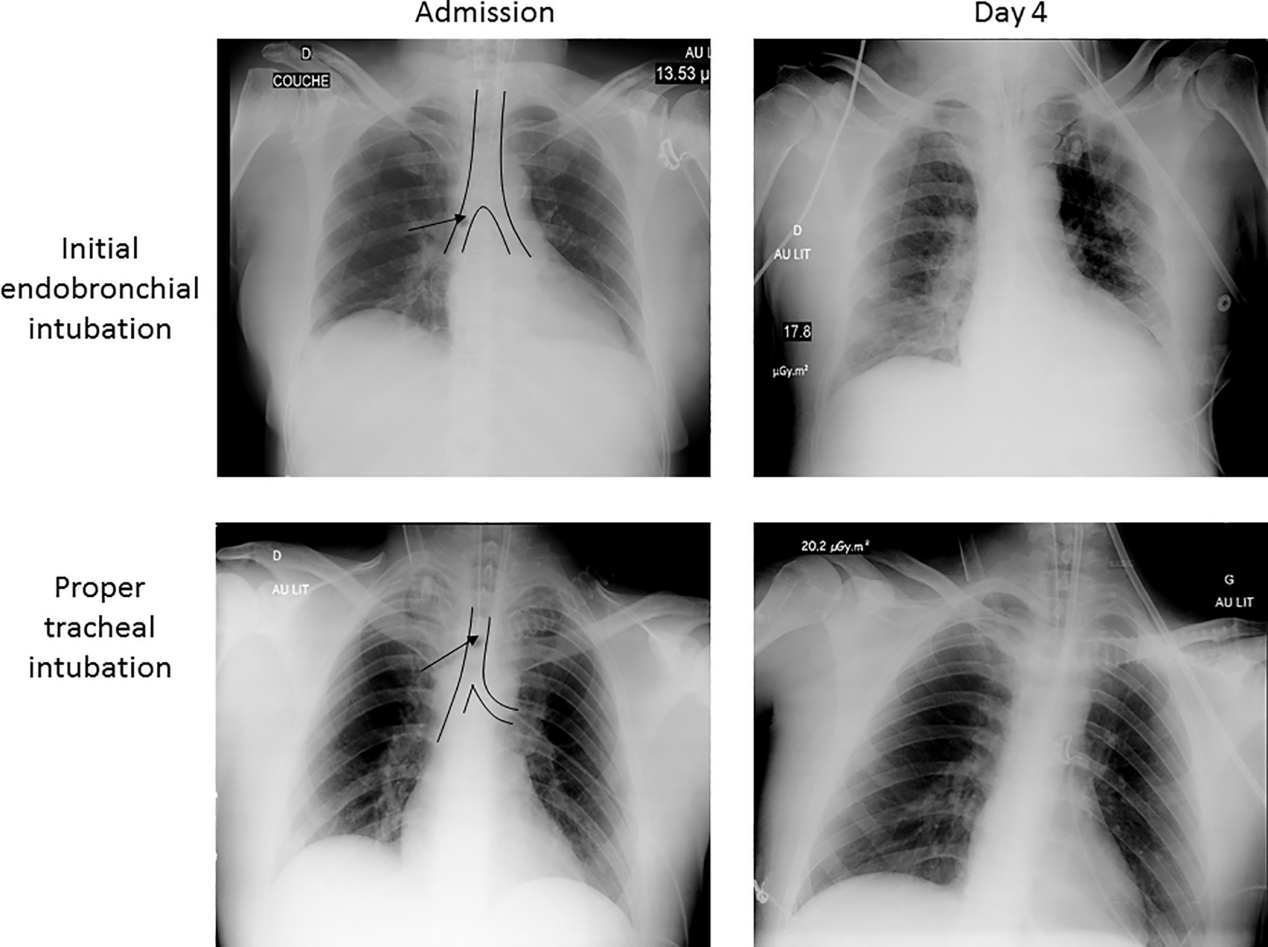
Chest X-ray obtained in a patient with an initial right endobronchial intubation and in another patient with initial proper tracheal intubation for comparison. The black arrow points to the tip of the endobronchial tube while the trachea and main right and left bronchi are underlined in black. On day 4, the patient with initial EBI developed basal lung infiltrates (predominant in the right base) compatible with VAP, whereas the patient with proper position of the tracheal tube on admission had an unremarkable chest X-ray.

During the study period, 398 patients underwent a field intubation and were subsequently admitted to our medical-surgical ICU. A total of 253 patients were excluded because they were early extubated (n = 104), died within the first two days following hospital admission (n = 90), had an underlying lung disease precluding accurate chest X-ray interpretation (n = 8), or for any other predefined reason (n = 51) (Fig 1). Finally, 145 patients were studied (median age: 56 IQR [41–69] years; 107 (74%) men; SAPS 2: 60 [45–67]). Reasons for field intubation were initial cardiorespiratory arrest (38%), severe polytrauma (49%), coma (11%), and acute respiratory failure (2%). Among patients intubated in the field for a cardiac arrest, 73% underwent targeted therapeutic hypothermia. Right-sided EBI was diagnosed in 33 patients (23%) (Fig 2), while no left-sided EBI was identified. Median duration of EBI was 143 [78–185] min.

No significant difference was observed between patients with EBI and their counterparts regarding age, gender, SAPS2, baseline diagnosis, antibiotic use in the last month, use of antibiotics for a reason different from VAP, fluid balance and transfusion (Table 1). All comorbidities were equally distributed among groups, except for immunosuppression, which was more common in the EBI group (36% vs. 16%; p = 0.011). Cardiac arrest was the main indication for intubation in the field in patients with EBI (55% vs. 33%: p = 0.025) (Table 1).

**Table 1:** Characteristics of the study population*.

	Total n = 145	Tracheal intubation n = 112 (77%)	Endobronchial intubation n = 33 (23%)	*p*
Gender M/F	107/38	82/30	25/8	0.770
Age	56 [41–69]	56 [38–68]	58 [50–70]	0.509
SAPS 2	60 [45–67]	58 [44–67]	62 [51–67]	0.414
**Comorbidities**				
Smoking	32 (22)	24 (21)	8 (24)	0.732
Alcohol consumption	19 (13)	13 (12)	6 (18)	0.325
Immunosuppression	30 (21)	18 (16)	12 (36)	0.011
Antibiotics during previous month	6 (4)	5 (5)	1 (3)	0.716
Antibiotics for other reasons	27 (19)	21 (19)	6 (18)	0.941
**Reason for field intubation**				
Cardiac arrest	55 (38)	37 (33)	18 (55)	0.025
Targeted therapeutic hypothermia	41/55 (73)	28/37 (76)	12/18 (67)	0.199
Trauma	71 (49)	60 (54)	11 (33)	0.041
Coma	16 (11)	13 (12)	3 (9)	0.685
Respiratory failure	3 (2)	2 (2)	1 (3)	0.659
**Characteristics of ICU stay**				
Mechanical ventilation (days)	8 [5–12]	7 [5–12]	8 [5–12]	0.968
Fluid balance (ml/24h)				
Day 4/Discharge	400 [-802–2543]	393 [-974–2538]	521 [-649–2886]	0.558
Date of VAP	236 [-1163–2952]	91 [-1263–1819]	1290 [-125–3308]	0.190
Transfusion (Units of packed red blood cells)				
Day 4/Discharge	0 [0–0]	0 [0–0.75]	0 [0–0]	0.315
Date of VAP	0 [0–0]	0 [0–1]	0 [0–0]	0.120
ICU stay (days)	10 [6–16]	10 [5–16]	10 [7–16]	0.771
Mortality	53 (37)	42 (38)	11 (33)	0.906

*: continuous variables are expressed as medians; numbers between parentheses are either interquartile ranges or percentages.

Abbreviations: SAPS: Simplified acute physiology score, ICU: Intensive care unit.

The overall rate of early VAP, late VAP and total VAP in our cohort was 20%, 17%, and 37% respectively, with a full agreement of adjudicators. Overall rate of early VAT, late VAT and total VAT was 7%, 10%, and 17%, respectively. Inadvertent EBI after field intubation showed a trend towards a higher proportion of early VAP (30% vs. 17%: p = 0.09), and of both early VAP and early VAT (39% vs. 23%: p = 0.065) (Table 2). The prevalence of late VAP and late VAT was similar between patients with EBI and with adequate tracheal intubation (Table 2). Mean time for the development of early VAP was also similar between groups (4 [IQR 3–4] vs. 3 [IQR 2–4] days for early VAP: p = 0.112 (Table 2). No significant correlation was found between EBI duration and early VAP development in univariate logistic regression (123 [110–180] min under EBI for patients without early VAP vs. 120 [66–187] min under EBI in patients with subsequent early VAP: r^2^ = 1; p = 0.933) (S1 Table).

**Table 2:** Ventilator associated respiratory tract infections.

	Total n = 145 Median [IQR] or n(%)	Tracheal intubation n = 112 Median [IQR] or n(%)	Endobronchial intubation n = 33 Median [IQR] or n(%)	*p*
Early VAP	29 (20)	19 (17)	10 (30)	0.09
Early VAP onset (days)	4 [3–4]	3 [2–4]	4 [3.3–4]	0.112
Early VAT	10 (6.9)	7 (6.3)	3 (9.1)	0.571
Total early VAP+VAT	39 (26.9)	26 (23.2)	13 (39.4)	0.065
Late VAP	24 (16.6)	18 (16.1)	6 (18.2)	0.774
Late VAP onset (days)	6 [6–9]	6.5 [6–9]	6 [6–7.5]	0.508
Late VAT	14 (9.7)	12 (10.7)	2 (3.1)	0.426
Total late VAP+VAT	38 (26.2)	30 (26.8)	8 (24.2)	0.770
Total VAP	53 (36.6)	37 (33.0)	16 (48.5)	0.105
Total VAT	24 (16.6)	19 (16.9)	5 (15.2)	0.805

Patients who developed early VAP had a similar proportion of immunosuppression than those who did not (28% vs. 19%: p = 0.305), but were more frequently intubated in the field for a cardiac arrest (55% vs. 34%: p = 0.032) and received more antibiotics for any other reasons than VAP (Table 3). Nevertheless, the association between cardiac arrest and early VAP was weighted down in the univariate analysis (p = 0.107) (S1 Table), and did not appear to be related to the use of targeted therapeutic hypothermia (p = 0.808) (Table 3 and S1 Table). The use of antibiotic therapy for any other reason than VAP was similar irrespective of tracheal tube position (19% vs. 18%: p = 0.941) (Table 1), but was only observed in the subset of patients who failed developing early VAP (23% vs. 0%: p = 0.004) (Table 3). Nevertheless, this association was not confirmed by the univariate analysis (p = 0.971) (S1 Table). The multivariate analysis failed to identify any factor which was independently associated with the development of early VAP (data not shown).

**Table 3:** Comparison between patients developing early VAP with those who did not.

	Total population n = 145	Early VAP n = 29	No early VAP n = 116	*p*
Gender M/F	107/38	24/5 (82.8)	83/33 (71.6)	0.219
Age	56 [41–69]	56 [49–70]	56 [40.3–68.3]	0.705
SAPS 2	60 [45–67]	58 [49–66]	61 [45.8–67.5]	0.926
**Comorbidities**				
Smoking	32 (22)	9 (31.1)	23 (19.8)	0.193
Alcohol consumption	19 (13)	3 (10.3)	16 (13.8)	0.623
Immunosuppression	30 (21)	8 (27.6)	22 (18.9)	0.305
Diabetes	24 (16.6)	8 (27.6)	16 (13.8)	0.074
Antibiotics during previous month	6 (4)	0 (0)	6 (5.2)	0.211
Antibiotics for other reasons	27 (19)	0 (0)	27 (23.3)	0.004
**Reason for field intubation**				0.116
Cardiac arrest	55 (38)	16 (55.2)	39 (33.6)	0.032
Targeted therapeutic hypothermia	41/55 (72.7)	12/16 (75)	28/39 (71.8)	0.808
Trauma	71 (49)	12 (41.4)	59 (50.9)	0.361
Coma	16 (11)	1 (3.4)	15 (12.9)	0.145
Respiratory failure	3 (2)	0 (0)	3 (2.6)	0.382
**Endobronchial intubation**	33 (22.8)	10 (34.5)	23 (19.8)	0.092
**Characteristics of ICU stay**				
Mechanical ventilation (days)	8 [5–12]	11 [7–13]	7 [5–12]	0.269
Fluid balance (ml/24h) at Day 4/Discharge	400 [-802–2543]	230 [-527–1109]	400 [-916–2553]	0.699
Transfusion (Units of packed red blood cells) Day 4/Discharge	0 [0–0]	0 [0–0]	0 [0–0]	0.787
ICU stay (days)	10 [6–16]	11 [8–18]	9 [5–16]	0.813
Mortality	53 (37)	13 (44.8)	40 (34.5)	0.301

Abbreviations: VAP: ventilator associated pneumonia; VAT: ventilator associated tracheo-bronchitis; IQR: interquartile range

Median length of ICU stay in the entire study cohort was 10 days [IQR: 6–16], and 37% of patients died in the ICU. There was no inter-group difference in the duration of mechanical ventilation (8 days [IQR 5–12] vs. 7 days [IQR 5–12]: p = 0.968), length of ICU stay (10 days [IQR 7–16] vs. 10 days [IQR 5–16]: p = 0.771) and ICU mortality (33% vs. 38%: p = 0.906), according to tracheal tube position (Table 1). Patients developing VAP irrespective of its delay of occurrence exhibited a longer length of ICU stay (16 [10–24] days vs. 8 [5–12] days: p<0.001) and remained during a longer period under mechanical ventilation (12 [8–17] days vs. 6 [4–9]: p<0.001) (S2 Table).

Microorganisms most frequently associated with VAP were methicillin-susceptible *Staphylococcus aureus* (MSSA) (19%), mainly in patients with EBI (38%), followed by *Streptococcus pneumoniae* (11%) and *Haemophilus influenzae* (8%) (S3 Table).

## Discussion

The present study showed that patients intubated in the field who present to the hospital with endobronchial intubation (EBI) have a trend towards an increase of early ventilator associated pneumonia (VAP) and both early VAP and ventilator associated tracheo-bronchitis (VAT) when compared to those with a proper positioning of the tube in the trachea, even though the difference fails to reach statistical significance presumably due to a lack of power.

EBI has been reported in 11 to 17% of field intubations [9,25], and in only 4 to 7% of hospital intubations [14,15,26,27]. Incidence of adverse events for field intubation is variable across studies. This is presumably related to a potential report bias in retrospective studies [28], to variable experience in operators performing and evaluating the adequacy of tracheal intubation [11,12,28], and to the criterion used to define EBI. When the definition of EBI is not restricted to the intubation of the main (right) bronchus but rather extends to the location of the tip of the tube less than 1 to 3 cm from the carina [15,20], its prevalence raises up to 21% [29]. In keeping with these results, the prevalence of EBI in the present study reached 23%.

Field intubation is a known independent risk factor for early VAP [1,30]. This could be related to an increased rate of aspiration of gastric content prior to [31] or during intubation [1], underlying diseases [6,27], and to the indication requiring field intubation. We purposely excluded patients with overt aspiration identified during direct laryngoscopy or on admission chest X-ray to assess more precisely the potential association between inadvertent EBI and subsequent development of early VAP or other respiratory tract infections. Although immunosuppression was more frequently encountered in patients with EBI compared to patients with proper tracheal intubation, it was not significantly associated with VAP irrespective of its delay of occurrence. Blunt trauma, head and neck trauma [31,32], multiple injury, cardiorespiratory arrest [30], impaired consciousness, and intra-abdominal diseases [1] have been described as independent risk factors for VAP. In the present study, most patients requiring field intubation were trauma patients as previously reported [9]. In this subset of patients, the tracheal tube was predominantly positioned properly and no association with VAP was observed, irrespective of its time of development. Interestingly, of all reasons for field intubation, patients who were successfully resuscitated for an out-of-hospital cardiac arrest exhibited the highest prevalence of EBI. This is presumably due to the inward displacement of the tracheal tube during resuscitation maneuvers, which indirectly validates the chosen definition of EBI in the present study. Whether cardiac arrest constitutes an independent risk factor for early VAP as previously suggested [30], or is associated with this infectious complication due to the higher frequency of EBI in this clinical setting could not be determined since both the univariate and multiple regression analysis were not informative. The administration of antibiotics for any other reason than VAP is a potential confounding factor in this type of clinical study, as reflected by its absence in our patients who developed early VAP. Nevertheless, antibiotic therapy was uniformly used in the present cohort, irrespective of initial tracheal tube location, and was unrelated to the occurrence of early VAP in the univariate analysis. Not surprisingly, the prevalence of late VAP was similar in patients with initial EBI and in those with a proper tracheal tube positioning since the development of this delayed infectious complication is multifactorial, thus minimizing the potential role of inadequate initial intubation.

VAP definition is a matter of debate and there is no gold standard [1,4,33–35]. However, the diagnostic criteria used by the adjudication committee in the present study failed to result in any discrepancy between the experts when they independently adjudicate VAP cases in our patients. The overall 37% rate of VAP and 20% rate of early VAP identified in the current cohort are in keeping with previously reported prevalence which ranges between 30% and 49% after prehospital intubation [6,7], and between 15% and 21% in ICU patients [3,5,8,21]. Bacterial isolates from patients with early respiratory tract infection consisted in Gram positive cocci (*S*. *aureus*, *S*. *pneumoniae*) and *H*. *influenzae* in two thirds of cases in the EBI group, as previously reported in patients with ICU-acquired early VAP [8,27,30]. In contrast, patients with an adequate tracheal intubation showed a more even distribution of pathogens among Gram-positive cocci, Gram-negative bacilli and poly-microbial isolates.

The main limitation of the present study is its retrospective and single-center design. This did not allow to control for factors such as compliance to bundle of VAP prevention and assessment of potential aspiration, characteristics of the secretions, degree of pulmonary recruitment and ventilator mode, which are known to influence the development of VAP. Nevertheless, standards of care did not change during the relatively short time of the study, its single-center design resulted in homogeneous pre-hospital and ICU management, and overt aspiration during direct laryngoscopy are routinely reported in medical charts. Similarly, the lack of uniformly accepted definition of both EBI and VAP is an obstacle for administrative database inquiry. In addition, the lack of power of the current study resulted solely in a trend towards more frequent early VAP (and VAT) in patients with initial EBI when compared to those with proper tracheal tube positioning, but precluded reaching statistical significance. Similarly, the stepwise multivariate logistic regression analysis could not determine if cardiac arrest constitutes an independent risk factor *per se* or due to its frequent association with EBI. Accordingly, the present results remain to be confirmed in larger, adequately powered, prospective studies. Finally, EBI and respiratory tract infections (i.e., VAP and VAT) currently lack of consensual diagnosis criteria. Nevertheless, the screening for potentially eligible patients was exhaustive during the study period and the diagnoses of EBI and respiratory tract infections were independently adjudicated by experienced intensivists.

In summary, this study showed a high rate of inadvertent EBI after field intubation which was associated with a statistically non-significant trend towards early-onset respiratory tract infection (VAP and VAT) increase. Since the present retrospective study was limited by a lack of power, these results warrant to be confirmed prospectively in a larger multicenter cohort with a special emphasis on patients who sustained a cardiac arrest.

## Supporting information

S1 Table Univariate logistic regression. (DOC)

S2 Table Comparison between patients developing VAP irrespective of its delay of occurrence with those who did not. (DOC)

S3 Table Microbiological documentation of Ventilator-Associated Tracheo-bronchitis and Ventilator-Associated Pneumonia. (DOC)

S1 Dataset Complete anonymized database including all data collected from the patients during the study. (XLS)
