# Supplementary material for: Is prehospital endobronchial intubation a risk factor for subsequent ventilator associated pneumonia? A retrospective analysis
Source: PLoS One. 2019 May 23;14(5):e0217466. doi: 10.1371/journal.pone.0217466 (PMC6532927; doi:10.1371/journal.pone.0217466)
Supplement: S1 Table — (DOC) [file pone.0217466.s001.doc]

**S1 Table. Univariate logistic regression**

|  | **Total population** | **Early VAP** | **Not early VAP** | ***p*** |
| --- | --- | --- | --- | --- |
|  | **n=145** | **n=29** | **n=116** |  |
| Gender M/F | 107/38 | 24/5 (82.8) | 83/33 (71.6) | 0.225 |
| Age | 56 [41-69] | 56 [49-70] | 56 [40.3-68.3] | 0.708 |
| SAPS 2 | 60 [45-67] | 58 [49-66] | 61 [45.8-67.5] | 0.921 |
| **Comorbidities** |  |  |  |  |
| Smoking | 32 (22) | 9 (31.1) | 23 (19.8) | 0.197 |
| Hospitalization during previous month | 6 (4.1) | 1 (3.5) | 5 (4.3) | 0.835 |
| Alcohol consumption | 19 (13) | 3 (10.3) | 16 (13.8) | 0.624 |
| Immunosuppression | 30 (21) | 8 (27.6) | 22 (18.9) | 0.308 |
| Diabetes | 24 (16.6) | 8 (27.6) | 16 (13.8) | 0.079 |
| Antibiotics during previous month | 6 (4) | 0 (0) | 6 (5.2) | 0.979 |
| Antibiotics for other reasons | 27 (19) | 0 (0) | 27 (23.3) | 0.971 |
| **Reason for field intubation** |  |  |  |  |
| Cardiac arrest | 55 (38) | 16 (55.2) | 39 (33.6) | 0.107 |
| Targeted therapeutic hypothermia | 41/55 (72.7) | 12/16 (75) | 28/39 (71.8) | 0.808 |
| Trauma | 71 (49) | 12 (41.4) | 59 (50.9) | 0.371 |
| Coma | 16 (11) | 1 (3.4) | 15 (12.9) | 0.302 |
| Respiratory failure | 3 (2) | 0 (0) | 3 (2.6) | 0.978 |
| **Endobronchial intubation** | 33 (22.8) | 10 (34.5) | 23 (19.8) | 0.097 |
| **Duration under EBI** | 121 [77.5-184.5] | 120 [66-187] | 123 [109.5-180] | 0.933 |
| **Characteristics of ICU stay** |  |  |  |  |
| Mechanical ventilation (days) | 8 [5-12] | 11 [7-13] | 7 [5-12] | 0.350 |
| ICU stay (days) | 10 [6-16] | 11 [8-18] | 9 [5-16] | 0.837 |
| Mortality | 53 (37) | 13 (44.8) | 40 (34.5) | 0.303 |
