# Supplementary material for: Is prehospital endobronchial intubation a risk factor for subsequent ventilator associated pneumonia? A retrospective analysis
Source: PLoS One. 2019 May 23;14(5):e0217466. doi: 10.1371/journal.pone.0217466 (PMC6532927; doi:10.1371/journal.pone.0217466)
Supplement: S2 Table — (DOC) [file pone.0217466.s002.doc]

**S2 Table.** **Comparison between patients developing VAP irrespective of its delay of occurrence with those who did not**

|  | **Total population** | **Total VAP** | **No VAP** | ***p*** |
| --- | --- | --- | --- | --- |
|  | **n=145** | **n=53** | **n=92** |  |
| Gender M/F | 107/38 | 45/8 | 62/30 | 0.021 |
| Age | 56 [41-69] | 58 [48-70] | 55 [38-67.5] | 0.596 |
| SAPS 2 | 60 [45-67] | 58 [47.8-66] | 61 [44.5-67] | 0.630 |
| **Comorbidities** | | | | |
| Smoking | 32 (22) | 15 (28.3) | 17 (18.5) | 0.169 |
| Alcohol consumption | 19 (13) | 6 (11.3) | 13 (14.1) | 0.629 |
| Immunosuppression | 30 (21) | 13 (24.5) | 17 (18.5) | 0.386 |
| Diabetes | 24 (16.6) | 10 (18.9) | 14 (15.2) | 0.569 |
| Antibiotics during previous month | 6 (4) | 3 (5.7) | 3 (3.3) | 0.485 |
| Antibiotics for other reasons | 27 (19) | 8 (15.1) | 19 (20.7) | 0.408 |
| **Reason for field intubation** | | | | 0.451 |
| Cardiac arrest | 55 (38) | 23 (43.4) | 32 (34.8) | 0.303 |
| Targeted therapeutic hypothermia | 41/55 (72.7) | 17/23 (73.9) | 23/32 (71.9) | 0.867 |
| Trauma | 71 (49) | 25 (47.2) | 46 (50) | 0.743 |
| Coma | 16 (11) | 5 (9.4) | 11 (12) | 0.216 |
| Respiratory failure | 3 (2) | 0 (0) | 3 (3.3) | 0.184 |
| **Endobronchial intubation** | 33 (22.8) | 16 (30.2) | 17 (18.5) | 0.105 |
| **Characteristics of ICU stay** | | | | |
| Mechanical ventilation (days) | 8 [5-12] | 12 [8-17] | 6 [4-9] | < 0.001 |
| Fluid balance (ml/24h) at Day 4/Discharge | 400 [-802 – 2543] | 230 [-694 – 3138] | 400 [-1185 - 2351] | 0.277 |
| Transfusion (Units of packed red blood cells) Day 4/Discharge | 0 [0 – 0] | 0 [0 – 0] | 0 [0 – 0] | 0.806 |
| ICU stay (days) | 10 [6-16] | 16 [10-24] | 8 [5-12] | < 0.001 |
| Mortality | 53 (37) | 19 (35.8) | 34 (37) | 0.894 |
