# Supplementary material for: Is prehospital endobronchial intubation a risk factor for subsequent ventilator associated pneumonia? A retrospective analysis
Source: PLoS One. 2019 May 23;14(5):e0217466. doi: 10.1371/journal.pone.0217466 (PMC6532927; doi:10.1371/journal.pone.0217466)
Supplement: S3 Table — (DOC) [file pone.0217466.s003.doc]

**S3 Table. Microbiological documentation of Ventilator-Associated Tracheo-bronchitis and Ventilator-Associated Pneumonia**

|  | **Total** | **Tracheal intubation** | **Endobronchial intubation** |
| --- | --- | --- | --- |
| **Total VAP** | **n=53** | **n=37** | **n=16** |
| *Staphylococcus aureus* | 11 (21%) | 4 (11%) | 7 (44%) |
| MSSA | 10 (19%) | 4 (11%) | 6 (38%) |
| MRSA | 1 (2%) | 0 (0%) | 1 (6%) |
| *Streptococcus pneumoniae* | 6 (11%) | 3 (8%) | 3 (19%) |
| *Haemophilus influenzae* | 4 (8%) | 3 (8%) | 1 (6%) |
| Other Gram-negatives | 19 (36%) | 15 (41%) | 4 (25%) |
| Poly-microbial | 13 (25%) | 12 (32%) | 1 (6%) |
| **Total VAT** | **n=24** | **n=18** | **n=6** |
| *Staphylococcus aureus* | 8 (33%) | 6 (33%) | 2 (33%) |
| MSSA | 7 (29%) | 5 (28%) | 2 (33%) |
| MRSA | 1 (4%) | 1 (6%) | 0 (0%) |
| *Streptococcus pneumoniae* | 1 (4%) | 1 (6%) | 0 (0%) |
| *Haemophilus influenzae* | 3 (13%) | 2 (11%) | 1 (17%) |
| Other Gram-negatives | 7 (29%) | 5 (28%) | 2 (33%) |
| Poly-microbial | 5 (21%) | 4 (22%) | 1 (17%) |

Abbreviations: MSSA: Methicillin-susceptible *Staphylococcus aureus*, MRSA: Methicillin-resistant *Staphylococcus aureus*, VAP: ventilator-associated pneumonia; VAT: ventilator-associated trachea-bronchitis
